# An Anisotropic Velocity Model for Microseismic Events Localization in Tunnels

**DOI:** 10.3390/s23104670

**Published:** 2023-05-11

**Authors:** Tong Shen, Songren Wang, Xuan Jiang, Guili Peng, Xianguo Tuo

**Affiliations:** 1 School of Information Engineering, Southwest University of Science and Technology, Mianyang 621000, China; 2 School of Control and Mechanical, Tianjin Chengjian University, Tianjin 300384, China; 3 School of Automation and Information Engineering, Sichuan University of Science and Engineering, Zigong 643000, China

**Keywords:** anisotropic, velocity model, microseismic, localization, active source, MLKNN

## Abstract

The velocity model is one of the main factors affecting the accuracy of microseismic event localization. This paper addresses the issue of the low accuracy of microseismic event localization in tunnels and, combined with active-source technology, proposes a “source–station” velocity model. The velocity model assumes that the velocity from the source to each station is different, and it can greatly improve the accuracy of the time-difference-of-arrival algorithm. At the same time, for the case of multiple active sources, the MLKNN algorithm was selected as the velocity model selection method through comparative testing. The results of numerical simulation and laboratory tests in the tunnel showed that the average location accuracy of the “source–station” velocity model was improved compared with that of the isotropic velocity and sectional velocity models, with numerical simulation experiments improving accuracy by 79.82% and 57.05% (from 13.28 m and 6.24 m to 2.68 m), and laboratory tests in the tunnel improving accuracy by 89.26% and 76.33% (from 6.61 m and 3.00 m to 0.71 m). The results of the experiments showed that the method proposed in this paper can effectively improve the location accuracy of microseismic events in tunnels.

## 1. Introduction

The construction and maintenance of ultra-deep-buried tunnels pose significant engineering and technical challenges [1,2]. Rockburst is among the pivotal factors that impact heavily on the safety of deep-buried tunnels. It can result in injuries to workers, damage to equipment, and prolongation of construction time, and in severe cases, it can even trigger earthquakes [3,4,5]. Hence, it is imperative to monitor and provide early warning systems for rockburst events in tunnels. Microseismic monitoring technology is an essential tool for the early detection and monitoring of rockbursts [6]. Unlike other monitoring technologies, microseismic monitoring technology is capable of describing the spatiotemporal distribution of microseismic events in the monitored area. Therefore, the precise location of microseismic events is of utmost importance for most of the rockburst prediction methods based on microseismic monitoring, which are currently reliant on the statistical features of these events [7,8,9].

Tunnel engineering construction projects typically follow a linear path, and microseismic events frequently occur in proximity to the tunnel face; most occur beyond the scope of the sensor array. Thus, compared to conventional microseismic monitoring techniques, locating microseismic events in tunnel engineering is challenging [10,11]. In microseismic event localization, the commonly used methods are based on interferometry and time difference of arrival (TDOA) [12,13,14,15]. In tunnel engineering, TDOA-based methods are utilized the most frequently. However, due to the presence of large-scale openings (the already excavated part of the tunnel), the precision of the velocity model is low, leading to a lower location accuracy for the traditional TDOA-based algorithm. Scholars generally agree on this issue [16,17]. Numerous scholars have conducted comprehensive research on optimizing velocity models in tunnels; for example, Feng et al. [18] proposed a sectional velocity model, which considers that each group of sensors has the same velocity model and jointly solves microseismic event parameters using particle swarm algorithms until accuracy requirements are met. While this velocity model is particularly useful when the layer and tunnel are nearly perpendicular to one another, it is less effective when they form a certain angle or when the layer distribution is more complex, resulting in inconsistencies in sensor group velocity [19]. In addition, it is essentially still an SSH algorithm, and the introduction of more unknown parameters will lead to instability in the solution process and increase computational complexity [20,21]. In order to generate velocity models under different geological conditions, Ma et al. [22] proposed four different equivalent velocity models. However, these equivalent models cannot generate arbitrary complex velocity models. Peng et al. [23] proposed a 3D anisotropic velocity model based on the grid, which fully considers the impact of the excavation area when establishing the tunnel velocity model. It sets the velocity model of the excavation area to 340 m/s and the velocity of other areas to be consistent, and it uses block location (BL) to determine the approximate position of microseismic events. It then uses the improved nonlinear iterative particle swarm optimization (NPSO) algorithm to obtain the optimal solution. The aforementioned methods can achieve good results to a certain extent, but when the local geological structure is complex, it is often difficult to obtain desirable efficiency and results.

As is widely recognized, anisotropic velocity models can provide a more accurate representation of the propagation of microseismic events, although their development can be complicated [24]. In this paper, we present a “source–station” velocity model for microseismic event localization in tunnels, which takes into account that the velocity from the source to each station is different, making the velocity model anisotropic. Additionally, we assume that the propagation paths from similar sources to stations are similar, meaning that they can share the same velocity model. To exploit this assumption, an active-source array was deployed near the tunnel face, and a classification algorithm was used to select the active source closest to the microseismic event for location purposes.

The key contributions of this paper are as follows: (1) By combining active-source technology with the assumption that similar sources have similar propagation paths, we constructed the “source–station” velocity model. (2) The “source–station” velocity model allows for the omission of wave-type discrimination; this not only simplifies the computation process but also enhances the precision of localization. (3) We propose a method for selecting velocity models in the presence of multiple active sources.

The second part of the paper delves into the details of the “source–station” velocity model and explains the need for selecting velocity models when multiple active sources are involved. In the third part, we conduct numerical simulations experiments and laboratory tests in a tunnel environment to validate the accuracy of our proposed velocity model. We compare the results of these experiments with those obtained using isotropic velocity models and sectional velocity models. Additionally, we compare the accuracy of different velocity model selection methods and determine that the MLKNN algorithm is the best choice for selecting velocity models. The last section concludes the paper.

## 2. Methodologies

### 2.1. Location Method Based on Arrival-Time Theory

Microseismic events localization based on arrival-time theory is the most widely used location method. The TDOA algorithm determines the parameters of the microseismic events by calculating the residual between the observed arrival time and the theoretical arrival time [25]. Suppose that *S* (*x*_0_, *y*_0_, *z*_0_, *t*_0_) represents the source parameters of the microseismic event, where (*x*_0_, *y*_0_, *z*_0_) represents the spatial location of the source *S* and *t*_0_ represents the initiation time of source *S*, *T_i_* (*x_i_*, *y_i_*, *z_i_*, *t_i_*) is the *i*-th station used to record the microseismic events, whose position (*x_i_*, *y_i_*, *z_i_*) is known, and the arrival time *t_i_* can be obtained through the arrival-time picking algorithm. The residual of station *i* can be described with Equation (1), where $t_{i}^{obs}$ represents the observed travel time from source to station *i*, while $t_{i}^{cal}$ represents the theoretical travel time from source to station *i*.

(1) $$\varepsilon =t_{i}^{obs}-t_{i}^{cal}$$

(2) $$t_{i}^{obs}=t_{i}-t_{0}$$

Suppose the velocity is isotropic, and the velocity value is *v*, $t_{i}^{cal}$ can be expressed as:

(3) $$t_{i}^{obs}=R_{i}/V$$

where $R_{i}$ represents the distance between the source *S* and the station *i*:

(4) $$R_{i}=\sqrt{{\left(x_{0}-x_{i}\right)}^{2}+{\left(y_{0}-y_{i}\right)}^{2}+{\left(z_{0}-z_{i}\right)}^{2}}$$

By eliminating the simultaneous equation of all station residuals (Equation (1)), Equation (5) can be derived as follows:

(5) $$f={\sum_{i=1}^{N}\left|t_{i}^{obs}-t_{i}^{cal}\right|}^{n}={\sum_{i=1}^{N}\left|(t_{i}-t_{0})-\frac{R_{i}}{V}\right|}^{n}$$

In Equation (5), *f* represents the absolute value of the sum of the time residuals between the observation time and the calculated time at all stations (L1 norm), *N* represents the number of sensors, and *n* represents the norm (usually 1 or 2, related to the choice of the L1 or L2 norm). When the number of data points was limited, *n* = 2 was too sensitive to input errors. Therefore, in subsequent calculations in this paper, *n* = 1 [26].

As described in the principle of the arrival-time-difference location algorithm, Equation (5) uses the velocity from the source to the station, and, thus, the velocity model is one of the key factors affecting the accuracy of the location. Previous research has also confirmed that events outside the station are more sensitive to velocity model errors, and microseismic events in tunnels usually occur outside the station range [27]. Therefore, for the location of microseismic events in tunnels, an accurate velocity model is crucial.

### 2.2. “Source–Station” Velocity Model in a Tunnel

**Figure 1:**
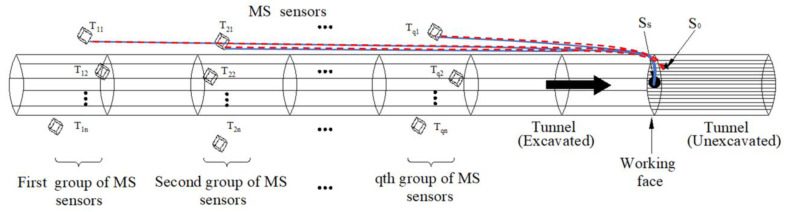
“Source–station” velocity model in tunnel. The solid black circle represents the location of the source, and the solid black pentagram represents the location of a microseismic event near the active source. The blue solid line represents the propagation path from the active source to the first station in group q, and the red dashed line represents the propagation path from the microseismic event to the corresponding station.

As shown in Figure 1, *S*_0_ represents the microseismic event to be located, and *S_S_* is the event induced by the nearby active source. The solid line (blue) in the figure represents the propagation path from *S_S_* to station *T_i_*_1_, and the dotted line (red) represents the propagation path from *S*_0_ to station *T_i_*_1_. We assumed that the propagation speeds *V_i_* from *S_S_* to *T_i_* are different, but due to the proximity of the two events, it can be assumed that the propagation paths of *S*_0_ and *S_S_* to each station were similar. Thus, when locating the unknown event *S*_0_, the velocity model *V_i_* determined by the active source *S_S_* could be used. Based on this idea, this paper proposes a “source–station” velocity model based on active sources. In this velocity model, the velocities from the source to each station are different, and there are no constraints between the velocities. Therefore, the “source–station” velocity model can be considered an anisotropic velocity model.

Since the “source–station” velocity model is determined by the active source and only makes use of the information of the initial arriving wave, it is not necessary to distinguish the type (P wave, S wave, or other) of the initial arriving wave when locating the events nearby. This not only makes the calculation simpler and more convenient but also reduces the error caused by misjudging the type of the initial arriving wave and improves the localization accuracy.

### 2.3. Location Method Using the “Source–Station” Velocity Model

The source parameters of the active source *S_S_* are (*x_s_*, *y_s_*, *z_s_*, *t_s_*), and the source parameters of the event *S*_0_ to be located are (*x*_0_, *y*_0_, *z*_0_, *t*_0_), where *t_i_* is the time when station *i* is first triggered by the source, and the equation for locating event *S*_0_ can be expressed using the following equation:

(6) $$f={\sum_{i=1}^{N}\left|(t_{i}-t_{0})-\frac{R_{i}{}^{0}}{V_{i}}\right|}^{n}$$

In Equation (6), *R_i_*^0^ represents the distance from event *S*_0_ to station *i*, which can be expressed by Equation (7), and *V_i_* is the velocity model determined by the active source *S_S_*, which can be expressed by Equation (8):

(7) $$R_{i}{}^{0}=\sqrt{{\left(x_{i}-x_{0}\right)}^{2}+{\left(y_{i}-y_{0}\right)}^{2}+{\left(z_{i}-z_{0}\right)}^{2}}$$

(8) $$V_{i}=\frac{R_{i}^{S}}{t_{i}-t_{S}}$$

In Equation (8), *R_i_^s^* represents the distance between event *S_S_* and station *i* and can be represented as follows:

(9) $$R_{i}{}^{0}=\sqrt{{\left(x_{i}-x_{S}\right)}^{2}+{\left(y_{i}-y_{S}\right)}^{2}+{\left(z_{i}-z_{S}\right)}^{2}}$$

If the active source is a controllable source, then its start time *t_s_* can be easily obtained through the microseismic events recording system.

If the active source is a source with an accurate start time that is difficult to directly obtain, such as a blast, human impact, etc., the TDOA method can be used to eliminate the effect of start time [28]. We can modify the residual function in Equation (6) to Equation (10), and Equation (11) can be used to express the “source–station” velocity model.

(10) $$f={\sum_{i-1}^{N-1}\left|(t_{i+1}-t_{i})-\frac{R_{i+1}^{0}-R_{i}^{0}}{V_{i}}\right|}^{n}$$

(11) $$V_{i}=\frac{R_{i+1}^{0}-R_{i}^{S}}{t_{i+1}-t_{i}},i\in [1,N-1]$$

## 3. Experiments and Discussions

### 3.1. Numerical Simulation in the Tunnel

**Figure 2:**
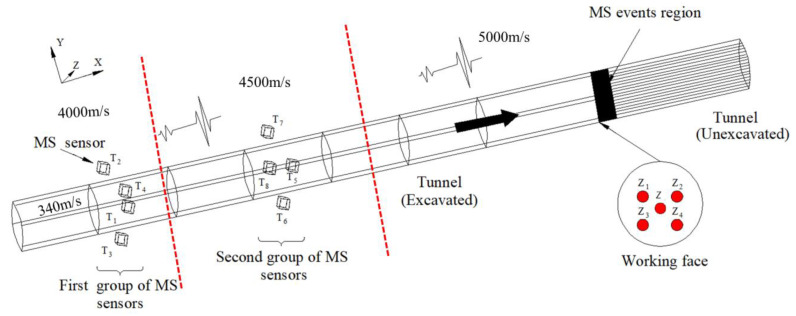
Simulation of microseismic monitoring in a tunnel. The eight cubes represent eight stations, which were placed in two groups. The five solid red circles represent five active sources (Z and Z1–Z4), which were set on the tunnel face. Among the five active sources, four (Z1–Z4) were used to establish the “source–station” velocity model, and one (Z) was used to establish the isotropic velocity model and the sectional velocity model. One hundred microseismic events were randomly distributed in the black zone. The red line represents the layered structure of the medium in the numerical simulations.

As shown in Figure 2, in this part of our research, we simulated the microseismic monitoring situation in the tunnel according to the method proposed by Feng et al. [18]. Eight stations were partially located within a circular tunnel with a diameter of twelve meters, and their coordinates are listed in Table 1. We placed five active sources on the tunnel face (working face in Figure 2). In order to better reflect the actual situation, one of the active sources was placed at the center of the tunnel face, which was used to establish the isotropic velocity model and the sectional velocity model. Their coordinates are listed in Table 2. One hundred microseismic events were randomly distributed after the tunnel face, assuming that the starting times of the active source and the microseismic events were both zero.

In the numerical simulation experiment, the modeling method proposed by Wang Yunhong et al. [29] was used for modeling. The source generation method used was the ellipsoid three-axis random generation method, and the time information generation method used was the 2.5D two-point fast ray tracing method. The distribution of the medium is shown by the red line in Figure 2, where the velocities of the medium were 4000 m/s, 4500 m/s, and 5000 m/s, and the velocity in the middle empty area was set to 340 m/s. Based on the coordinates of the microseismic sensors and of the 100 groups of microseismic events, and the velocities of each layer, the travel time information of the 100 groups of microseismic events was obtained. The positions and travel times of the random events can be found in Appendix A.

In this paper, a simulated annealing algorithm was used to locate 100 random events. The parameter settings for the simulated annealing algorithm were as follows: the initial search coordinate was (0, 0, 100), the upper limit of the search range for *x* and *y* was 10 and the lower limit was −10; the upper limit of the search range for *z* was 150 and the lower limit was 90; max iterations was 1000; the function tolerance was 1 × 10^−6^; and the temperature update function was an exponential temperature update. The results of the isotropic velocity model, the sectional velocity model, and the “source–station” velocity model were compared. The isotropic velocity model and the sectional velocity model were calculated using the active source Z, while the “source–station” velocity model was calculated using the active sources Z1–Z4. After the calculation, the isotropic velocity model was found to be 4695.84 m/s. The sectional velocity model used the method described in the literature. The two groups of velocities were 4613.00 m/s and 4902.64 m/s, respectively. In the “source–station” velocity model, all velocity models generated by the four active sources were brought into the calculation. The three velocity models are shown in Table 3.

**Figure 3:**
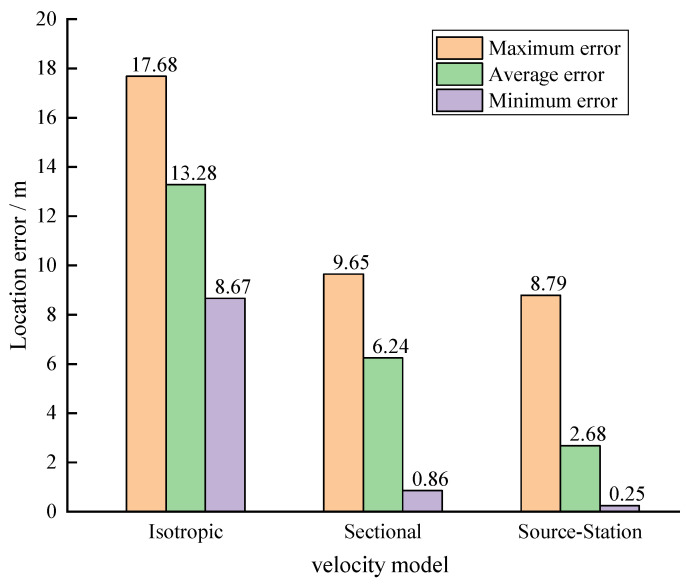
Location errors with different velocity models.

As shown in Figure 3, under the same conditions, the location results using different velocity models indicated that the average location error using the isotropic velocity model was the largest, with an average location error of 13.28 m. The average location error of the sectional velocity model was 6.24 m, reducing the location error by 53%, which was similar to Feng’s results [18]. When using the “source–station” velocity model, the average location error was the smallest, at only 2.68 m, which was significantly smaller than the other two velocity models. It reduces the location error by 57.05% compared with the sectional velocity model and by 79.82% compared with the isotropic velocity model, greatly improving the localization accuracy.

### 3.2. Selection of the “Source–Station” Velocity Model

During prior tunnel simulation experiments, four groups of active sources were established, resulting in relatively favorable outcomes for the localization. However, the calculation of the velocity model was determined from the active source situated close to the microseismic event, based on the assumption of a “source–station” velocity model. As the actual event–source distance was unknown, a methodology to determine the velocity model from multiple active sources had to be employed. For this study, the model and data depicted in Figure 2 were utilized to compare and contrast the effectiveness of the MLKNN algorithm, the KNN algorithm, the SVM algorithm, and ANN for selecting the optimal velocity model.

The dataset consisted of 100 sets of microseismic events from eight stations, and their arrival times and velocity models. In this case, we needed to select the best velocity model for each microseismic event from four possible velocity models. For each microseismic event, the eight arrival times were used as data feature values, and the best velocity model, serving as the class label, exhibited the minimum localization error.

**Figure 4:**
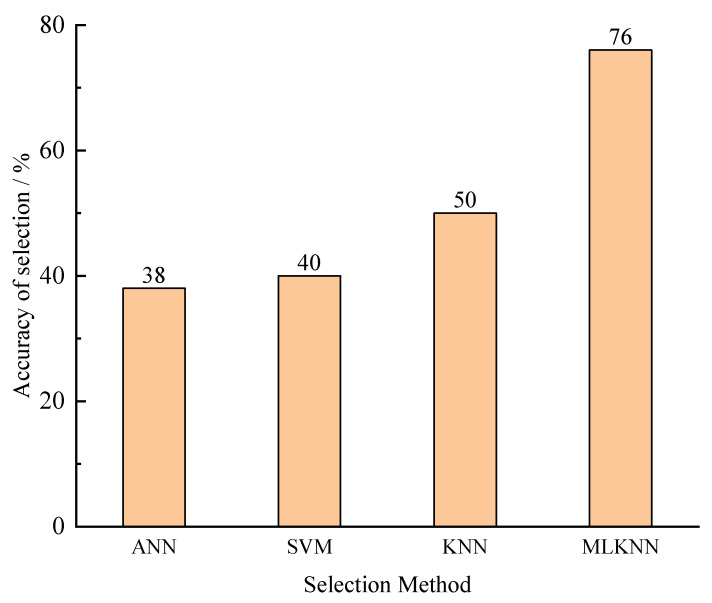
Comparison results obtained by different selection methods.

MLKNN used eight feature values, a label, and a second label (another velocity model with a localization error within 0.5 m, if it existed. If it did not exist, the second label was the same as the first one) as input, while KNN, SVM, and ANN used eight feature values and a label as input. Out of these 100 microseismic events, 50 sets of data were used for training, and 50 sets were used for testing. The comparison results are shown in Figure 4. MLKNN’s accuracy was 76%, KNN’s accuracy was 50%, SVM’s accuracy was 40%, and ANN’s accuracy was 38%. MLKNN exhibited a good selection performance with small sample sizes.

**Figure 5:**
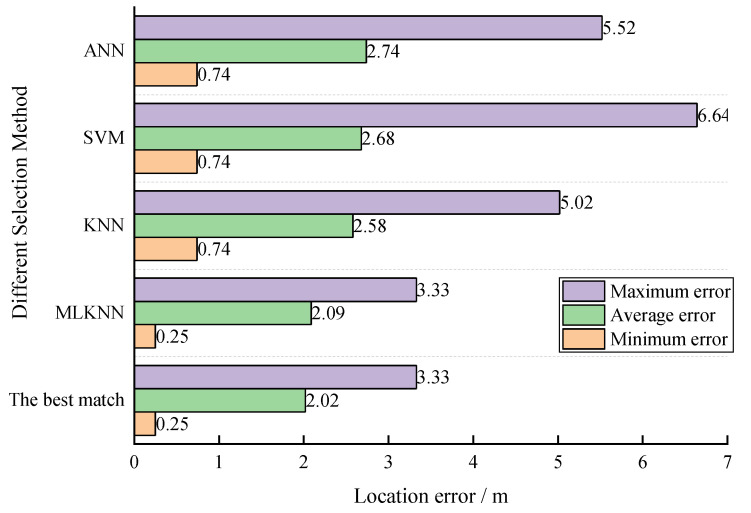
Comparison of location errors for different velocity model selection methods. The best match was the velocity model that exhibited the minimum localization error during the experiment.

We compared the localization errors of the optimal velocity model selected by four different methods with the actual localization error of the best velocity model, as shown in Figure 5. In the simulation test, despite the lower selection accuracy rate of 76% with the MLKNN algorithm, the location error was still relatively close to the best match.

### 3.3. Tests in the Tunnel Laboratory

**Figure 6:**
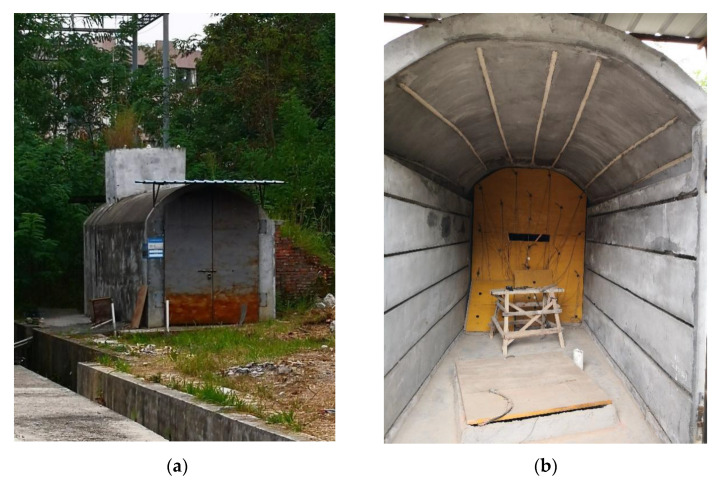
The tunnel laboratory of the School of Environment and Resources at Southwest University of Science and Technology. (**a**) Tunnel laboratory appearance; (**b**) tunnel laboratory structure.

The laboratory tests were carried out in the tunnel laboratory of the School of Environment and Resources at Southwest University of Science and Technology in Mianyang, Sichuan Province. The tunnel laboratory is a brick and concrete structure which is approximately 2.6 m wide, 8 m long, and 3 m high, as shown in Figure 6. In the tests, a self-developed data collection device and 14 Hz omnidirectional sensors were used, with a geophone sensitivity of 80 V/m/s and a data sampling rate of 10 kHz. The microseismic events were realized through manual striking, and a geophone was placed near the striking point. The waveform of the geophone and each station were cross-correlated to calculate the travel time of the seismic wave.

**Figure 7:**
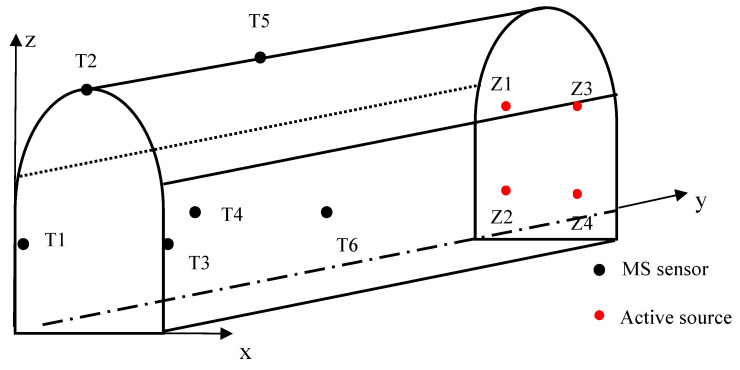
Distribution of stations (MS sensors) and active sources in the tunnel laboratory. The black dots represent the stations (T1–T6), and the red dots represent the active sources.

In the test, seven geophones (T1–T7) were deployed, among which T1–T6 acted as station sensors and T7 was placed at the source striking point. There were four active-source events (Z1–Z4) that were used to generate the models, and 10 microseismic events (S1–S10) that were yet to be located. Most of the microseismic events in the tunnel occurred near the roof; therefore, all the microseismic events in the test were distributed outside the station range. The spatial distribution of the sensors and active sources are shown in Figure 7, and the coordinates of the stations, active sources, and microseismic events are listed in Table 4, Table 5 and Table 6.

Unlike the numerical simulation experiments, in order to increase fairness in the comparison, the isotropic velocity model, the sectional velocity model, and the “source–station” velocity model were generated by the same set of active sources, giving four sets of velocity models, as presented in Table 7. As drilling holes inside the tunnel laboratory is not allowed, geophones were attached to the laboratory walls using stone adhesives. This may have caused some coupling issues and, consequently, the velocities determined may not be entirely accurate. However, since both the active sources and the events were triggered under these conditions, the coupling issues did not affect the location results of the microseismic events.

**Figure 8:**
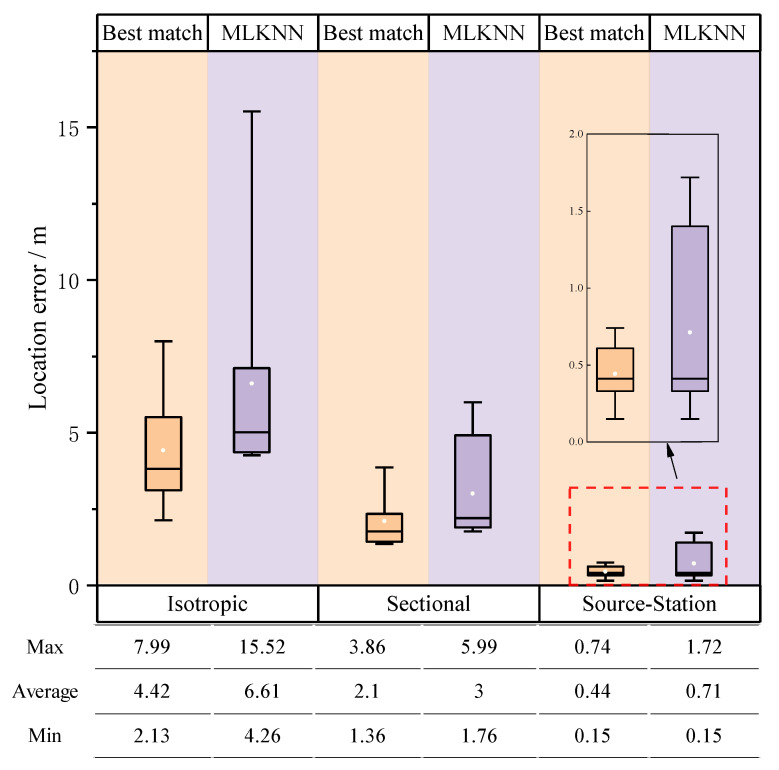
For each type of velocity model, the MLKNN algorithm was used to select the velocity model, and the location error of the selected velocity model was compared with that of the best-match velocity model. The best match was the velocity model that exhibited the minimum localization error during the experiment. The white dot in the box represents the average value of location error.

A simulated annealing method was employed for the localization of ten microseismic events. The previous experiments had demonstrated the satisfactory performance of the MLKNN method in the selection process. With the implementation of MLKNN, we obtained the localization errors for different velocity models and the best-match localization errors for each velocity model. A comparison of these results is illustrated in Figure 8. From the figure, we can observe the same pattern as in the previous numerical simulation experiments. The isotropic velocity model had the largest localization error, followed by the sectional velocity model, and the “source–station” velocity model had the lowest.

It is worth noting that even though the average localization errors for the isotropic velocity model and the sectional velocity model were 6.61 m and 3.00 m, respectively, which may seem insignificant, such localization errors are still considered unacceptable when taking into account the overall size of the tunnel laboratory.

## 4. Conclusions

In this paper, a “source–station” velocity model for microseismic event localization in tunnels was proposed. This velocity model is an anisotropic velocity model, where the velocity from the source or event to each station is different and there is no constraint between them. The establishment of this velocity model was simple and effective. Additionally, since events that are close in distance have similar propagation paths (the idea of double-difference localization), when using this velocity model for microseismic event localization, it is not necessary to determine the phase type, but only to identify the arrival time, reducing the localization error caused by incorrect phase identification. Therefore, this method can accurately reflect the propagation law of microseismic waves from a certain point and its surrounding area to the rock cave and stations, and this velocity model is suitable for use in the localization of microseismic events in tunnels.

The results of the tunnel numerical simulation experiment showed that the adoption of the “source–station” velocity model significantly improved the accuracy of microseismic events localization compared with the isotropic velocity model and the sectional velocity model, with the average localization error reducing by 79.82% (from 13.28 m to 2.68 m) and by 57.05% (from 6.24 m to 2.68 m), respectively. In the laboratory experiment, the results were similar to the numerical simulation results, with the average localization error reducing by 89.26% (from 6.61 m to 0.71 m) and by 76.33% (from 3.00 m to 0.71 m), compared to the isotropic velocity model and the sectional velocity model, respectively. We also discussed the selection of velocity models in the case of multiple active sources, and the localization results showed that the selection of velocity models was necessary. Although the current selection method (MLKNN) still has deficiencies, based on the excellent performance of the “source–station” velocity model after incorporating the selection of velocity models based on the MLKNN algorithm into the process, the average localization error in the simulation results increased by 0.07 m from the best-match result, which was 2.02 m, and still better than the best results of the isotropic velocity model and the sectional velocity model. In the laboratory test, the average localization error increased by 0.27 m from the best result, which was 0.44 m, and was also better than the best results of the isotropic velocity model and the sectional velocity model.

In the current approaches to locating microseismic events in tunnels, the “source–station” velocity model and the multisource selection method based on the MLKNN algorithm are reliable and effective. This method is also applicable to microseismic monitoring scenarios where the stations are far from the microseismic events.

The establishment and application of the “source–station” velocity model in practical engineering can be seen in [30], but due to construction periods and conditions, it is currently not possible to verify the selection of the “source–station” velocity model through field testing. In future work, the authors will continue to search for relevant experimental conditions to improve the experimental results. Additionally, the method of selecting the velocity model when there are multiple active sources will be further optimized to improve the accuracy of location of microseismic events in tunnels.

## Figures and Tables

**Table 1:** Coordinates of the microseismic stations (MS sensors).

Coordinates (m)	Microseismic Stations (MS Sensors)							
	1	2	3	4	5	6	7	8
*x*	0	8	−8	1	0	8	−8	−1
*y*	8	0	0	−9	8	0	0	−10
*z*	2	0	0	5	42	40	40	27

**Table 2:** Coordinates of the active sources.

Coordinates (m)	Active Sources				
	Z1	Z2	Z3	Z4	Z
*x*	2	−4	−3	3	0
*y*	3	2	−4	−3	0
*z*	102	102	102	102	102

**Table 3:** Different velocity models.

Type of Velocity Model	Velocity (m/s)							
Isotropic Velocity	4695.84							
Sectional Velocity Model	First Group: 4613.00				Second Group: 4902.64			
Source–station velocity model-1	4614.91	4601.06	4601.19	4637.19	4941.81	4926.21	4926.3	4846.82
Source–station velocity model-2	4614.94	4601.27	4601.02	4637.19	4941.84	4926.34	4926.19	4846.79
Source–station velocity model-3	4615.15	4601.24	4601.07	4636.98	4941.94	4926.35	4926.2	4846.66
Source–station velocity model-4	4615.1	4601.04	4601.24	4636.98	4941.92	4926.2	4926.32	4846.67

**Table 4:** Coordinates of microseismic stations (MS sensors).

Coordinates (m)	Microseismic Stations (MS Sensors)					
	1	2	3	4	5	6
*x*	0.00	1.00	2.00	0.00	1.30	2.00
*y*	0.50	0.60	0.40	3.74	4.50	3.85
*z*	1.50	2.70	1.60	1.35	2.65	1.42

**Table 5:** Active-source coordinates.

Coordinates (m)	Active Source			
	1	2	3	4
*x*	0.75	0.75	1.50	1.50
*y*	8.00	8.00	8.00	8.00
*z*	1.60	0.40	1.60	0.40

**Table 6:** Coordinates and travel time of MS events.

MS Source	Coordinates (m)			Travel Time of the Events (s)					
	*x*	*y*	*z*	1	2	3	4	5	6
1	1.10	8.00	1.70	0.02450	0.01125	0.00825	0.00725	0.01700	0.03025
2	2.00	6.52	1.63	0.03330	0.00700	0.00400	0.00350	0.00130	0.00050
3	2.00	6.12	1.39	0.03100	0.00550	0.00225	0.00125	0.00200	0.00175
4	0.00	7.67	1.40	0.01400	0.02375	0.03625	0.00400	0.01650	0.02550
5	1.45	8.00	1.37	0.02575	0.01175	0.00725	0.01000	0.01550	0.00180
6	2.00	7.18	1.70	0.03600	0.00750	0.00425	0.00400	0.00150	0.00225
7	1.30	8.00	1.00	0.02125	0.01175	0.00775	0.00775	0.00500	0.00050
8	2.00	6.88	1.31	0.03400	0.00375	0.00350	0.00350	0.00125	0.00325
9	0.00	6.98	1.49	0.01400	0.02375	0.03625	0.00400	0.01650	0.02550
10	1.23	8.00	0.54	0.02125	0.01125	0.00775	0.00575	0.00650	0.00100

**Table 7:** Different velocity models in the tunnel laboratory.

Type of Velocity Model	Velocity (m/s)					
Isotropic velocity-1	387.83					
Isotropic velocity-2	565.48					
Isotropic velocity-3	502.25					
Isotropic velocity-4	1007.23					
Sectional velocity model-1	First group: 500.98			Second group: 220.51		
Sectional velocity model-2	First group: 995.65			Second group: 559.37		
Sectional velocity model-3	First group: 516.41			Second group: 459.92		
Sectional velocity model-4	First group: 1172.79			Second group: 697.92		
Source–station velocity model-1	314.09	564.94	993.82	525.18	2052.93	1422.26
Source–station velocity model-2	346.24	674.19	1005.81	305.42	763.1	424.05
Source–station velocity model-3	294.2	638.13	761.64	952.27	236.1	2091.94
Source–station velocity model-4	255.45	1350.49	881.19	512.8	980.15	860.53

## Data Availability

The data presented in this study are available on request from the corresponding author.

## Appendix A

**Table A1:** Location and Travel Times of the 100 Microseismic Events.

Events	Location (m)			Travel Time of the Events (ms)							
	*x*	*y*	*z*	1	2	3	4	5	6	7	8
1	7	5	102	21.7314	22.1966	22.4316	21.1734	12.2387	12.6283	12.9879	15.8662
2	9	4	120	25.0277	25.4568	25.7069	24.4018	15.5361	15.8796	16.2307	19.0641
3	−5	1	102	21.7485	22.348	22.1797	21.0686	12.2654	12.8606	12.6022	15.6615
4	10	−5	120	25.179	25.4672	25.7448	24.2795	15.7501	15.8943	16.2837	18.9043
5	10	7	104	22.1385	22.5875	22.9144	21.6424	12.6679	13.0328	13.5249	16.3751
6	−8	−3	103	22.0465	22.6259	22.3604	21.2281	12.6253	13.1861	12.7821	15.7884
7	6	2	114	23.9186	24.3584	24.5356	23.2411	14.4221	14.7784	15.0341	17.8793
8	−10	7	116	24.3075	25.051	24.7627	23.8172	14.8213	15.6106	15.2003	18.4344
9	4	9	110	23.1404	23.7173	23.8401	22.7059	13.6209	14.1783	14.3582	17.3952
10	−2	5	103	21.8648	22.4811	22.4146	21.3231	12.3447	12.9668	12.8652	15.959
11	4	−9	110	23.4211	23.7173	23.8401	22.3828	14.036	14.1783	14.3582	16.9611
12	−10	6	124	25.7577	26.4702	26.2028	25.2185	16.2653	17.0021	16.6326	19.8187
13	4	1	108	22.8252	23.2769	23.4028	22.1218	13.3304	13.7018	13.889	16.7483
14	−10	−3	102	21.9052	22.518	22.1829	21.0916	12.5079	13.1186	12.6071	15.652
15	6	−4	106	22.5803	22.9166	23.1094	21.699	13.1478	13.3436	13.6324	16.3116
16	0	2	103	21.8892	22.4219	22.4219	21.2335	12.3826	12.8764	12.8764	15.8513
17	4	4	114	23.8811	24.3806	24.4988	23.2717	14.3672	14.8106	14.9812	17.9114
18	4	−9	109	23.2425	23.5364	23.6606	22.2011	13.8612	13.9987	14.1813	16.7797
19	−8	−7	101	21.7992	22.3108	22.0397	20.832	12.4454	12.9015	12.4853	15.3735
20	−3	10	119	24.7717	25.4542	25.3701	24.3522	15.249	15.9509	15.8324	19.0015
21	−10	7	109	23.0419	23.8033	23.4936	22.5717	13.5638	14.3897	13.9353	17.2003
22	−3	6	102	21.6832	22.3343	22.2335	21.1832	12.1633	12.8398	12.6851	15.8273
23	7	10	122	25.3516	25.892	26.0825	24.9052	15.8428	16.3432	16.6082	19.5938
24	4	8	121	25.1378	25.6928	25.803	24.6393	15.6159	16.1375	16.2918	19.2977
25	2	5	122	25.3168	25.8578	25.9124	24.7297	15.7937	16.2954	16.3718	19.3561
26	9	0	122	25.4312	25.8064	26.052	24.6839	15.9549	16.2234	16.5659	19.3207
27	1	−2	101	21.5969	22.0439	22.078	20.792	12.1316	12.4919	12.5447	15.3873
28	2	−4	104	22.1876	22.5864	22.6522	21.3108	12.743	13.0313	13.1312	15.8982
29	−1	−5	110	23.2899	23.7259	23.6951	22.3938	13.843	14.191	14.1457	16.9599
30	4	−2	114	23.9599	24.3695	24.4878	23.1568	14.4821	14.7945	14.9653	17.7596
31	7	−2	113	23.8104	24.1739	24.3826	23.0017	14.3483	14.5927	14.895	17.6247
32	4	−7	113	23.8971	24.23	24.3492	22.9318	14.4745	14.6743	14.8468	17.5147
33	−9	−5	108	23.0094	23.568	23.2858	22.129	13.6058	14.1322	13.7151	16.6772
34	−8	10	105	22.2851	23.0749	22.8165	21.9296	12.7949	13.672	13.2854	16.5989
35	−2	6	112	23.4951	24.1157	24.0554	22.9663	13.971	14.5912	14.5034	17.5965
36	−1	10	107	22.5834	23.2582	23.2265	22.2043	13.0582	13.7636	13.7165	16.8862
37	3	−5	124	25.8205	26.2112	26.2917	24.9366	16.3529	16.6443	16.7559	19.5169
38	8	9	111	23.3686	23.8829	24.1255	22.9232	13.8713	14.3357	14.6885	17.6367
39	2	10	114	23.8585	24.4767	24.5356	23.4467	14.3342	14.9493	15.0341	18.1253
40	−10	2	100	21.4546	22.1572	21.8142	20.8272	12.0132	12.766	12.2358	15.426
41	−1	−6	102	21.8797	22.2924	22.2588	20.9335	12.4686	12.7756	12.724	15.4967
42	6	−3	122	25.4416	25.8167	25.9806	24.6096	15.9695	16.2378	16.4667	19.2141
43	−9	6	107	22.6643	23.4003	23.1152	22.1635	13.1808	13.9736	13.5503	16.787
44	2	5	111	23.3182	23.8638	23.9247	22.7505	13.7965	14.3078	14.3969	17.3914
45	6	3	111	23.3647	23.818	24.0007	22.7229	13.8655	14.2405	14.5076	17.3724
46	−4	7	107	22.5955	23.2701	23.1431	22.1184	13.0767	13.7812	13.592	16.7596
47	6	3	124	25.7211	26.1801	26.341	25.0658	16.2142	16.6011	16.8242	19.698
48	−8	−9	111	23.6457	24.1255	23.8829	22.6359	14.278	14.6885	14.3357	17.1714
49	5	5	122	25.3351	25.8347	25.9712	24.7429	15.8195	16.263	16.4537	19.3841
50	9	6	107	22.6643	23.1152	23.4003	22.1266	13.1808	13.5503	13.9736	16.8317
51	−4	6	122	25.3229	25.9593	25.8501	24.7763	15.8023	16.4371	16.2846	19.3883
52	9	5	119	24.8405	25.283	25.5354	24.2465	15.3471	15.7093	16.0649	18.9171
53	10	−9	109	23.3245	23.5248	23.834	22.2741	13.9817	13.9814	14.4343	16.8982
54	−10	0	112	23.6444	24.2919	23.9912	22.9351	14.1906	14.8459	14.4096	17.5073
55	1	−9	118	24.8388	25.1938	25.2221	23.8283	15.4225	15.659	15.6992	18.3894
56	0	4	118	24.5927	25.1494	25.1494	23.9847	15.0713	15.5961	15.5961	18.6002
57	8	−6	118	24.812	25.1103	25.3369	23.8818	15.3844	15.5406	15.8611	18.4907
58	5	−4	121	25.2715	25.6454	25.7833	24.4104	15.805	16.0708	16.2642	19.0059
59	−6	−1	118	24.6837	25.2531	25.0827	23.9324	15.2017	15.7429	15.5013	18.5006
60	−3	−8	123	25.7144	26.1439	26.0628	24.7523	16.2776	16.622	16.5093	19.3013
61	9	−9	118	24.9102	25.1512	25.4055	23.8873	15.5235	15.5987	15.9575	18.4951
62	2	5	108	22.7731	23.3202	23.3832	22.2116	13.252	13.7664	13.8599	16.8574
63	−2	3	111	23.3337	23.9095	23.8485	22.7071	13.8196	14.3747	14.2854	17.3152
64	8	0	121	25.2358	25.6237	25.8442	24.4899	15.7545	16.0404	16.3492	19.1213
65	0	−8	112	23.732	24.1082	24.1082	22.7393	14.3186	14.5802	14.5802	17.2989
66	5	9	117	24.4202	24.9773	25.1202	23.9614	14.9029	15.4285	15.6315	18.6407
67	−6	−9	109	23.262	23.7108	23.5248	22.2417	13.89	14.2549	13.9814	16.7797
68	−7	−7	100	21.6048	22.0997	21.8595	20.6322	12.2474	12.6781	12.3067	15.1746
69	−10	9	123	25.5741	26.3288	26.0594	25.1252	16.0815	16.8775	16.5046	19.7479
70	5	−1	112	23.589	23.9968	24.1476	22.8159	14.1094	14.4179	14.6376	17.4318
71	0	6	112	23.4912	24.0818	24.0818	22.9585	13.9653	14.5419	14.5419	17.5965
72	−10	−10	115	24.4243	24.9187	24.628	23.3987	15.0692	15.5008	15.0866	17.931
73	−10	−4	120	25.1571	25.7371	25.4594	24.3237	15.7192	16.2728	15.8833	18.8733
74	−1	8	118	24.5792	25.2071	25.1787	24.0972	15.0518	15.6779	15.6377	18.7384
75	−4	−9	109	23.2425	23.6606	23.5364	22.2173	13.8612	14.1813	13.9987	16.7599
76	6	8	123	25.5183	26.045	26.2071	25.0117	16.0032	16.4845	16.7096	19.6776
77	10	10	115	24.1295	24.628	24.9187	23.6999	14.6455	15.0866	15.5008	18.426
78	−7	8	119	24.8039	25.5136	25.3174	24.3305	15.2949	16.0343	15.7579	18.9545
79	−4	−2	108	22.8761	23.4057	23.2798	22.086	13.4069	13.8933	13.7062	16.6572
80	0	−6	114	24.0347	24.4435	24.4435	23.1106	14.5907	14.9015	14.9015	17.6763
81	5	1	101	21.5677	21.9981	22.1682	20.8664	12.0859	12.4207	12.6837	15.51
82	1	3	116	24.2386	24.7673	24.7962	23.6	14.7216	15.2068	15.2483	18.2151
83	7	−10	109	23.3089	23.5403	23.7571	22.2295	13.9589	14.0044	14.3224	16.8267
84	−3	8	120	24.9499	25.603	25.5195	24.4663	15.4255	16.0853	15.9681	19.098
85	−1	−10	103	22.1932	22.5391	22.506	21.1065	12.8491	13.0549	13.0046	15.6566
86	1	−9	109	23.2278	23.5685	23.5996	22.1919	13.8395	14.046	14.0917	16.7537
87	4	10	107	22.5986	23.1938	23.3205	22.2095	13.0813	13.6677	13.8559	16.9171
88	8	7	112	23.5497	24.0337	24.2741	23.0352	14.0516	14.4717	14.8204	17.7284
89	−7	5	124	25.7186	26.3786	26.191	25.1444	16.2106	16.876	16.6163	19.7417
90	1	−1	104	22.1187	22.5844	22.6173	21.3516	12.6375	13.0281	13.0782	15.9512
91	−8	−4	109	23.1473	23.706	23.4575	22.2994	13.7206	14.2478	13.8818	16.8526
92	−10	9	102	21.7774	22.5927	22.2588	21.404	12.3103	13.231	12.724	16.0695
93	4	−5	120	25.1052	25.4777	25.5891	24.2145	15.646	15.9091	16.0659	18.8013
94	9	6	111	23.3879	23.8409	24.1142	22.841	13.8999	14.2742	14.6721	17.5357
95	6	−8	109	23.2297	23.5082	23.6944	22.2183	13.8424	13.9569	14.2309	16.8131
96	2	−5	106	22.5732	22.9579	23.0222	21.6643	13.137	13.4058	13.5023	16.2459
97	1	−9	122	25.5562	25.9167	25.944	24.5556	16.1301	16.3777	16.4158	19.1164
98	7	0	114	23.9571	24.3519	24.5586	23.2133	14.478	14.769	15.067	17.846
99	−10	−1	120	25.1017	25.7241	25.4463	24.3587	15.641	16.2548	15.8648	18.9214
100	3	6	104	22.0465	22.5957	22.6943	21.5279	12.5261	13.0454	13.1948	16.1965
